# Aging and chronic administration of serotonin-selective reuptake inhibitor citalopram upregulate Sirt4 gene expression in the preoptic area of male mice

**DOI:** 10.3389/fgene.2015.00281

**Published:** 2015-09-16

**Authors:** Dutt Way Wong, Tomoko Soga, Ishwar S. Parhar

**Affiliations:** Brain Research Institute, School of Medicine and Health Sciences, Monash University MalaysiaSelangor, Malaysia

**Keywords:** serotonin, aging, reproduction, sirtuins, cognition

## Abstract

Sexual dysfunction and cognitive deficits are markers of the aging process. Mammalian sirtuins (SIRT), encoded by *sirt 1-7* genes, are known as aging molecules which are sensitive to serotonin (5-hydroxytryptamine, 5-HT). Whether the 5-HT system regulates SIRT in the preoptic area (POA), which could affect reproduction and cognition has not been examined. Therefore, this study was designed to examine the effects of citalopram (CIT, 10 mg/kg for 4 weeks), a potent selective-serotonin reuptake inhibitor and aging on SIRT expression in the POA of male mice using real-time PCR and immunocytochemistry. Age-related increases of *sirt1, sirt4, sirt5, and sirt7* mRNA levels were observed in the POA of 52 weeks old mice. Furthermore, 4 weeks of chronic CIT treatment started at 8 weeks of age also increased *sirt*2 and *sirt*4 mRNA expression in the POA. Moreover, the number of SIRT4 immuno-reactive neurons increased with aging in the medial septum area (12 weeks = 1.00 ± 0.15 vs. 36 weeks = 1.68 ± 0.14 vs. 52 weeks = 1.54 ± 0.11, *p* < 0.05). In contrast, the number of sirt4-immunopositive cells did not show a statistically significant change with CIT treatment, suggesting that the increase in sirt4 mRNA levels may occur in cells in which *sirt4* is already being expressed. Taken together, these studies suggest that CIT treatment and the process of aging utilize the serotonergic system to up-regulate SIRT4 in the POA as a common pathway to deregulate social cognitive and reproductive functions.

## Introduction

Aging of the central nervous system deregulates homeostatic mechanisms responsible for sexual behavior (Davidson et al., 1983), feeding (Weindruch et al., 2001), sleep (Nakamura et al., 2011) and cognition (Barrientos et al., 2012). Sexual dysfunction and cognitive loss are prominent markers of the aging process. The preoptic area (POA) is involved in the hypothalamic-pituitary-gonadal (HPG) axis for the control of reproduction (Larsson and Heimer, 1964). Gonadotropin-releasing hormone (GnRH) is a pivotal molecule synthesize by neurons in the POA that regulates the release of gonadotropins (LH, luteinizing hormone and FSH, follicular stimulating hormone) that are important for reproduction and reproductive behaviors (Tsutsumi and Webster, 2009). The POA including the GnRH neurons receive serotonergic (5-hydroxytrypramine, 5-HT) innervations (Van De Kar and Lorens, 1979; Jennes et al., 1982). Pharmacological manipulations and lesions of the serotonergic system has a negative tone on reproduction (Verma et al., 1989; Kondo and Yamanouchi, 1997; Olivier et al., 2011) and cognitive function (Sibille et al., 2007). During aging, sex steroid deprivation shifts the homeostasis of the HPG axis which results in increase circulating LH and GnRH levels (Chakravarti et al., 1976). Furthermore, increases in LH are associated with decline in cognitive performance (Casadesus et al., 2007). Moreover, an age-related decline in the serotonergic system also leads to cognitive dysfunction (Meltzer et al., 1998). The POA is involved in social cognition (Driessen et al., 2014). Hence, to understand the mechanism of reproductive aging and cognitive loss, it is important to examine the serotonergic system in the POA during aging.

The family of seven sirtuin (SIRT) proteins is involved in the aging mechanism, which may include reproductive aging (Duan, 2013). Sirt activity is governed by its co-activator, nicotinamide adenine dinucleotide (*nad*), inhibitor nicotinamide (*nam*) and the intermediary conversion enzyme nicotinamide mononucleotide adenylyltransferase (*nmnat-1*) (Denu, 2005). All seven SIRT proteins are expressed in the brain (Dali-Youcef et al., 2007). SIRT proteins are involved in energy balance, reproduction and in brain aging (Duan, 2013). SIRT4 controls glutamate metabolism through glutamate dehydrogenase (Haigis et al., 2006), overexpression of which alters synaptic activity similar to serotonin-depleted models (Michaelis et al., 2011). In addition, serotonin1b (5-HT1b) receptor knockout mice, up-regulate *sirt5* in adult male mice, causing early onset of brain aging (Sibille et al., 2007).

Whether the 5-HT system regulates SIRT in the POA, which could affect the HPG axis, reproduction and cognition has not been examined. Treatment with citalopram (CIT), a potent selective-serotonin reuptake inhibitor, shows deficits in sexual behavior in adult mice (Soga et al., 2010) and sexual dysfunction in humans(Montejo et al., 2001), a condition that mimics aging, which has decreased 5-HT synthesis (Hussain and Mitra, 2000). CIT is extremely selective for its transporter, biosynthetic enzymes and receptors and is used pharmacologically to increase endogenous 5-HT levels at the synapse, although, chronic treatment decreases 5-HT synthesis (Moret and Briley, 1996; Bezchlibnyk-Butler et al., 2000; Stenfors et al., 2001). Therefore, this study was design to examine the effect of CIT and aging on *sirt* mRNA and SIRT expression in the POA of male mice using real-time PCR and immunocytochemistry respectively.

## Materials and methods

### Animals

Male C57BL/6N mice (CLEA Japan, Inc Tokyo, Japan) aged 12 weeks (weeks) (*n* = 33), 36 weeks (*n* = 8), and 52 weeks (*n* = 16) were maintained under standard conditions at the animal facility of the Brain Research Institute, Monash University Malaysia. These conditions include constant temperature (22°C) and lighting (12 h light/12 h dark cycle with lights on from 12:00 a.m.) with food and water available *ad libitum*. All procedures were approved by Animal Ethics Committee of Monash University (SOBSB/MY/2010/45) and were accordance with the Guidelines for the Care and Use of Animals by Monash University.

### Chronic citalopram treatment

Mice were administered with CIT [10 mg/kg body weight (BW) in 50 μl, C7861, Sigma-Aldrich, Singapore; *n* = 15] or 50 μl vehicle (distilled water; *n* = 15) daily at 9 a.m. by intraperitoneal (i.p) injections for 4 weeks beginning at 8 weeks of age until 12 weeks of age. Mice were used for two studies; gene expression study and immunocytochemical study. For the gene expression study [12 weeks (vehicle), *n* = 9 and 12 weeks CIT, *n* = 9] using the POA and compared with gene expressions of intact aged mice (36 weeks, *n* = 8 and 52 weeks, *n* = 10). For the immunocytochemical study, the POA of adult mice (12 weeks, *n* = 6, 12 weeks CIT, *n* = 6) and mid-age group (52 weeks, *n* = 6) was used. In this experiment, 52 weeks C57BL/6 male mice represented the reproductive aging model. This strain of mice at 52 weeks begin to exhibit declining male fertility (Fox et al., 2006) characterized by an increase in abnormal spermatozoa leading to ejaculatory disorders (Fabricant and Parkening, 1982), decrease in pheromone production (Wilson and Harrison, 1983), and a decrease in sexual arousal (Bronson and Desjardins, 1982). Additionally, at 52 weeks old these mice begin to experience age-related cognitive decline (Pettan-Brewer et al., 2013).

### Real-time PCR quantification of *sirt1-7, nam* and *nmnat-1* in the POA

**Figure 1:**
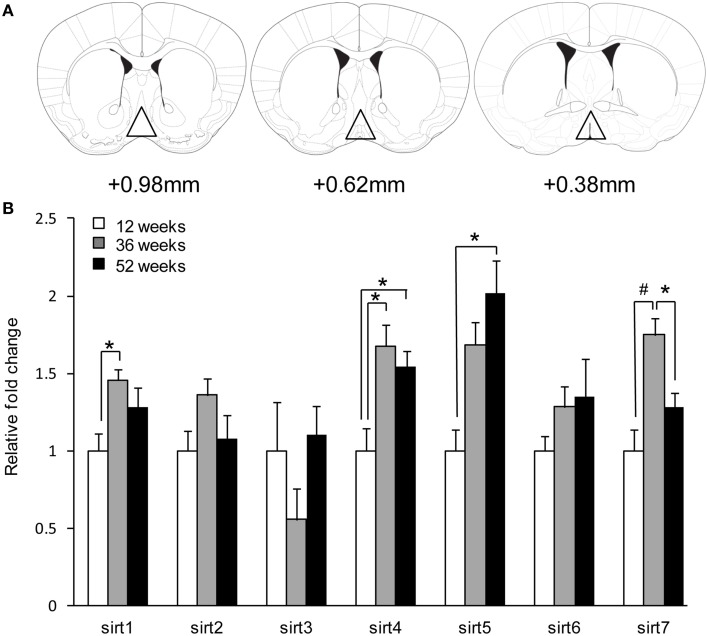
**Aging induces *sirt* gene expression in the preoptic area**. **(A)** Representative brain sections depicting areas dissected for gene expression studies in the POA. **(B)** Quantitative real-time PCR revealed differential *sirt* expression changes. Data are expressed as mean ± SEM. Statistical analysis was carried out using one-way analysis of variance (12 weeks control, *n* = 9; 36 weeks, *n* = 8; 52 weeks, *n* = 10). ^*^P < 0.05 and ^#^*P* < 0.01 vs. control.

At various ages (12weeks, 12weeks CIT, 36weeks, and 52weeks), animals were deeply anaethesized with an i.p. injection of ketamine xylazine (4.5 mg/kg/BW) followed by rapid removal of the brain and snap frozen. The POA (bregma +0.98 to +0.26, 8–11 sections/brain) was cut on a cryostat (60 μm/section) and each section further dissected with a sterile blade under naked eye (Figure 1A). Total RNA from these tissues was extracted using TRIzol (Invitrogen, Carlsbad, CA, USA) and transcribed using High Capacity Transcription Kit (Applied Biosystems, Foster City, CA, USA) according to manufacturer's protocols. Quantitative real-time PCR was performed on a ABI 7300 (Applied Biosystems Foster City, CA, USA) using 2X Power SYBR Green PCR mix (Applied Biosystems), and 0.2M primers for *sirt1-7, nam*, and *nmnat-1* (Supplementary Table 1) in a final volume of 10 μl. The resulting PCR products were validated using an ABI PRISM 310 Genetic Analyzer and Sequence Analysis Software (Applied Biosystems) and ran on a 2.5% agarose gel with ethidium bromide used for visualization.

### SIRT4 immunocytochemistry

Male mice at 12 weeks (control, *n* = 6; 12 weeks CIT, *n* = 6) and 52 weeks of age (control, *n* = 6) were anaesthesized with an i.p. injection of ketamine xylazine (4.5 mg/kg) and perfused transcardially with 4% paraformaldehyde (PFA) in 0.1M phosphate buffer (pH 7.5). The brains were removed, postfixed for 4 h in 4% PFA, and cryoprotected in 30% sucrose overnight. The POA (bregma +0.98 to +0.26 mm) was then sectioned at 30 μm in the coronal plane into three equal series. A set of sections was incubated for 20 min at 60°C in sodium citrate (pH 6) to unmask antigen epitopes. Prior to incubation in blocking solution (0.25% bovine serum albumin and 1.0% Triton X in 0.1M PBS) for 1 h with tissues were washed twice in 0.1M phosphate buffer saline (PBS) for 10 min each. Subsequently, the same 0.1M PBS wash was conducted on tissues prior to every incubation step. Sections were then incubated for 48 h at 4°C in a polyclonal goat anti-SIRT4 antibody (ab10140, Abcam, USA) at 1:500 dilution in blocking solution. Next, tissues were incubated with biotinylated rabbit anti-goat (Vector Laboratories, Burlingame, CA, USA) at 1:300 dilution in blocking solution for 1 h. Following this, sections were incubated in avidin-peroxidase (1:100 Vector Laboratories) and immunoreactive signal was observed using nickel-enhanced 3,3′-diaminobenzidine hydrochloride (Sigma). Sections were thoroughly washed in PBS and mounted on SuperFrost Plus slides (Fisher Scientific, Pittsburgh, PA, USA), air-dried, dehydrated in ethanol followed by xylene. Finally, the slides were coverslipped with DPX mounting medium.

### Double-label immunofluorescence

Coronal POA sections (30 μm) from male mice at 12 weeks (*n* = 3) were used for double immunohistochemistry with NeuN or GFAP and antisera to SIRT4 as described above. Tissue sections were incubated in Alexa Fluor 488 Anti-Goat (1:200, A11055, Molecular Probes) for 1 h and then incubated with either polyclonal rabbit antibody against GFAP (1:500, G9269, Sigma) or mouse monoclonal antibody against NeuN (1:500, MAB377, Millipore-Chemicon, Billerica, MA, USA) for 24 h. Sections incubated with antisera to GFAP were incubated with Alexa Fluor 594 Anti-Rabbit (1:200, A11012, Molecular Probes) while those with antisera to NeuN were incubated with Alexa Fluor 594 Anti-Mouse (1:200, A11005, Molecular Probes). After a final 0.1M PBS wash, sections were mounted on SuperFrost plus slides (Fisher Scientific) and coverslipped with VECTASHIELD mounting medium (H-1000, Vector Laboratories).

### Absorption test and SIRT4 antibody specificity

The SIRT4 antibody employed in this study recognizes amino acids 302–314 at the C-terminal. For testing antibody specificity, two procedures were carried out. Firstly, an absorption test was carried out using intact 12 weeks mice POA sections (*n* = 3) at 1:500 SIRT4 antisera pre-absorbed overnight with SIRT4 protein (AB23185; amino acids 302–314, 1 μg/ml, Abcam) in immunohistochemical procedures. Secondly, the primary SIRT4 antibody was omitted from the primary incubation solution. Both pre-absorbed and omission of SIRT4 antisera did not produce any immunoreactive staining.

### SIRT4 immunoreactive analysis

POA sections were viewed using bright-field microscopy (Nikon Eclipse 50i) and images were captured in TIF format (Nikon, Tokyo, Japan). The distribution of SIRT4 immunoreactivity was mapped throughout the mouse forebrain and brainstem. Cell counts for SIRT4 immunoreactive cells in the POA of 12 weeks, 12 weeks CIT treated, and 52 weeks (90 μm apart) were carried out using Image Pro Plus (Media Cybernetics Incorporation, Bethesda, USA). The POA consisting of the medial septum (MS), organum vasculosum of the lamina terminalis (OVLT) and the anterior hypothalamic area (AHA) were defined as per unit area of 500,000 pixels, 400,000 pixels, and 50,000 pixels respectively using Image Pro Plus. For each animal, two anatomically matched tissues per area were captured and used for cell counts. Cell counts were carried out by a researcher blind to the treatment and age. A single SIRT4 immunostained cell in a single focal plane was quantified by Image Pro Plus as 120 pixels. Therefore, any clusters of immunoreactive cells quantified by Image Pro Plus were divided by 120 pixels to obtain cell number counts, only immunostained cells with a full size (120 pixels) nuclei were counted in each image of the POA to make adjustments for double counts, in order to obtain true SIRT4 positive cell numbers. Data is expressed as mean number of identifiable SIRT4-immunoreactive cells.

### Double-label immunofluorescence analysis

The co-localization of NeuN or GFAP with SIRT4 immunoreactive cells was viewed with a 20X objective lens under a fluorescent microscope (Nikon Eclipse 90i, Tokyo, Japan). Sections were viewed using a Texas red filter to observe NeuN and GFAP labeled cells while a fluorescein-isothiocyanate filter was used to observe SIRT4 immunolabeled cells. Images of co-localized cells, SIRT4/NeuN and SIRT4/GFAP, were further captured using a laser scanning confocal microscope (C1si, Nikon) and software (NIS Elements AR v4.0, Nikon). The images were captured at 1024 pixel density using a 20X objective and 3X digital zoom function at every 1.25 μm interval to cover the entire neuronal volume.

### Statistical analysis

Statistical analysis for the effect of age and treatment (CIT) on sirtuin and NAD was carried out using one-way Analysis of variance (ANOVA) PASW statistic software (Version 17.0 Chicago, IL USA), followed with *post-hoc* analysis, Tukey's test, for comparison of multiple age groups. The effect of CIT was further analyzed using unpaired Student's *t*-test. Significant main interactions from One-Way ANOVA were further analyzed using Student's *t*-test. Statistical analysis for the effect of age and CIT on SIRT4 protein expression was carried out using Two-Way ANOVA using age and region (MS, OVLT and AHA) as factors followed by *T*-test to determine significance. Data are presented as means ± SEM. Significant difference was considered when *p* < 0.05.

## Results

### Effects of aging on *sirt* expression in the POA

Aging did not alter *sirt2, sirt3*, and *sirt6* gene expression in the POA of male mice. There was an increase in *sirt4* and *sirt5* mRNA expression in the POA during aging [*sirt4*; 12 weeks 1.00 ± 0.15 vs. 36 weeks 1.68 ± 0.14 vs. 52 weeks 1.54 ± 0.11, *F*_(2, 27)_= 7.20, *p* < 0.01] and [*sirt5*; 1.00 ± 0.14 vs. 36 weeks 1.69 ± 0.15 vs. 52 weeks 2.02 ± 0.21, *F*_(2, 27)_= 10.40, *p* < 0.01] (Figure 1B). *Post-hoc* analysis revealed an increase in *sirt4* (12 weeks vs. 52 weeks; *p* < 0.05) and *sirt5* (12 weeks vs. 52 weeks; *p* < 0.05). *sirt1* and *sirt7* showed an increase only during 36 week [sirt1; 12 weeks 1.00 ± 0.11 vs. 36 weeks 1.46 ± 0.07 vs. 52 weeks 1.28 ± 0.12, *F*_(2, 27)_ = 4.93, *p* < 0.05 and *sirt7;* 12 weeks 1.00 ± 0.14 vs. 36 weeks 1.75 ± 0.11 vs. 52 weeks 1.28 ± 0.09, *F*_(2, 27)_ = 11.26, *p* < 0.01] (Figure 1B). *Post-hoc* analysis for *sirt7 r*evealed a decrease at 52 weeks compared to 36 weeks of age (36 weeks vs. 52 weeks; *p* < 0.05).

### Effects of CIT on *sirt* expression in the POA

**Figure 2:**
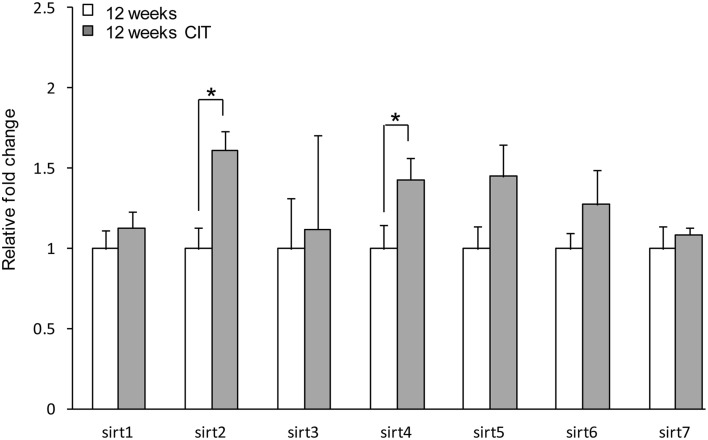
**Chronic citalopram (CIT) treatment up-regulates *sirt2* and *sirt4* in the preoptic area**. CIT was administered at 10 mg/kg/bodyweight daily in male mice for a duration of 4 weeks leading to relative mRNA changes in *sirt2* and *sirt4* expression in the POA. There was no difference in sirt3, sirt5, sirt6, or sirt7 mRNA levels in the POA after CIT treatment. Data are expressed as mean ± SEM. Statistical analysis was carried out using one-way analysis of variance (12 weeks control, *n* = 9; 12 weeks CIT, *n* = 9). ^*^P < 0.05 vs. control.

Chronic CIT treatment induced an increase in *sirt2* (12 weeks 1.00 ± 0.13 vs. 12 weeks CIT 1.61 ± 0.12, *p* < 0.05) and *sirt4* (12 weeks 1.00 ± 0.15 vs. 12 weeks CIT 1.43 ± 0.13, *p* < 0.05) expression in the POA (Figure 2). There was no difference in *sirt3, sirt5, sirt6, and sirt7* levels in the POA after CIT treatment (Figure 2).

### Effects of aging and CIT on *nampt* and *nmnat-1* expression in the POA

**Figure 3:**
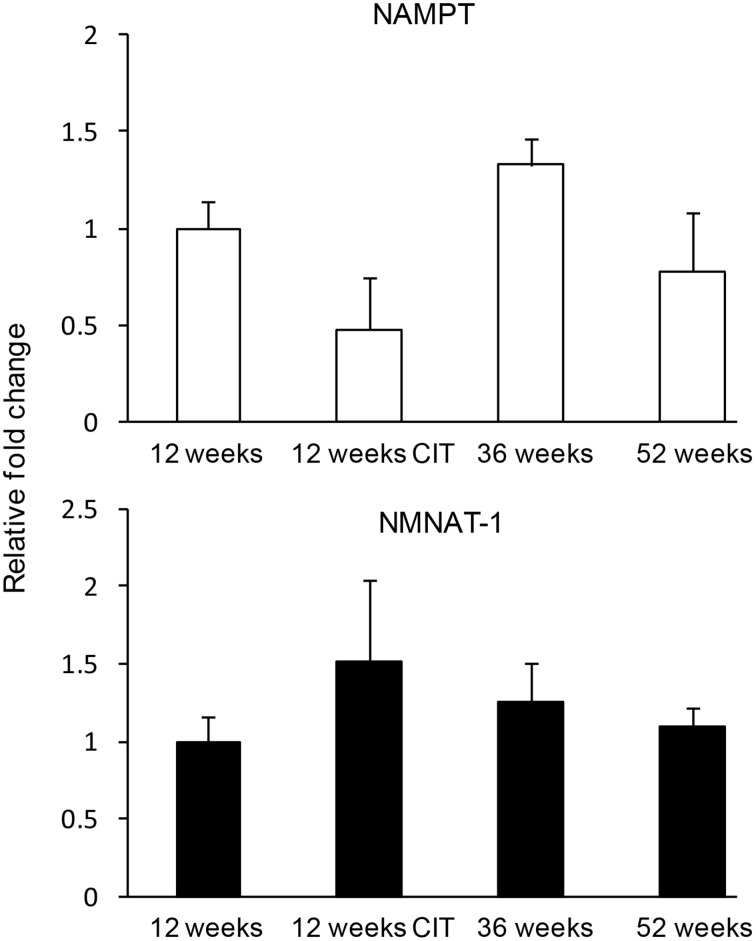
**Nicotinamide adenine dinucleotide (NAD) biosynthethic enzymes were not affected by chronic citalopram (CIT) treatment or aging**. CIT was administered at 10 mg/kg/bodyweight daily in male mice for 4 weeks duration. Data are expressed as mean ± SEM. Statistical analysis was carried out using one way analysis of variance (12 weeks control, *n* = 9; 12 weeks CIT, *n* = 9) aging animals used were 36 weeks (*n* = 8) and 52 weeks (*n* = 10).

CIT and aging had no effect on *nam* and *nmnat-1* expression in the POA (Figure 3).

### Effects of aging and CIT on SIRT4 protein in the POA

**Figure 4:**
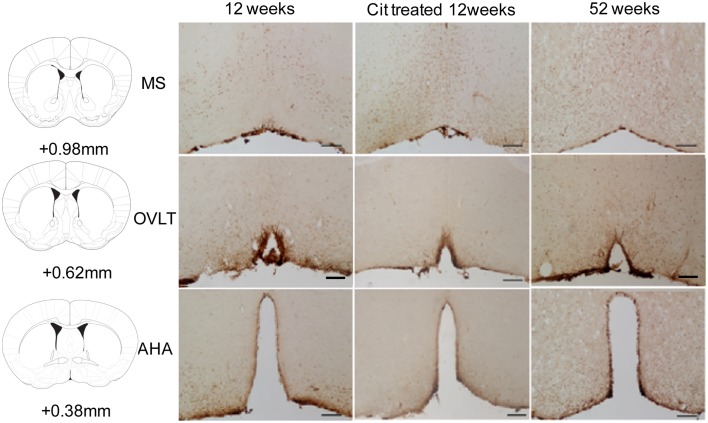
**Representative images of SIRT4 immunoreactivity in the medial septum (MS), organum vasculosum of the lamina terminalis (OVLT) and anterior hypothalamic area (AHA) from control, citalopram (CIT) treated and mid-aged samples (52 weeks)**. Scale bar = 100 μm.

**Figure 5:**
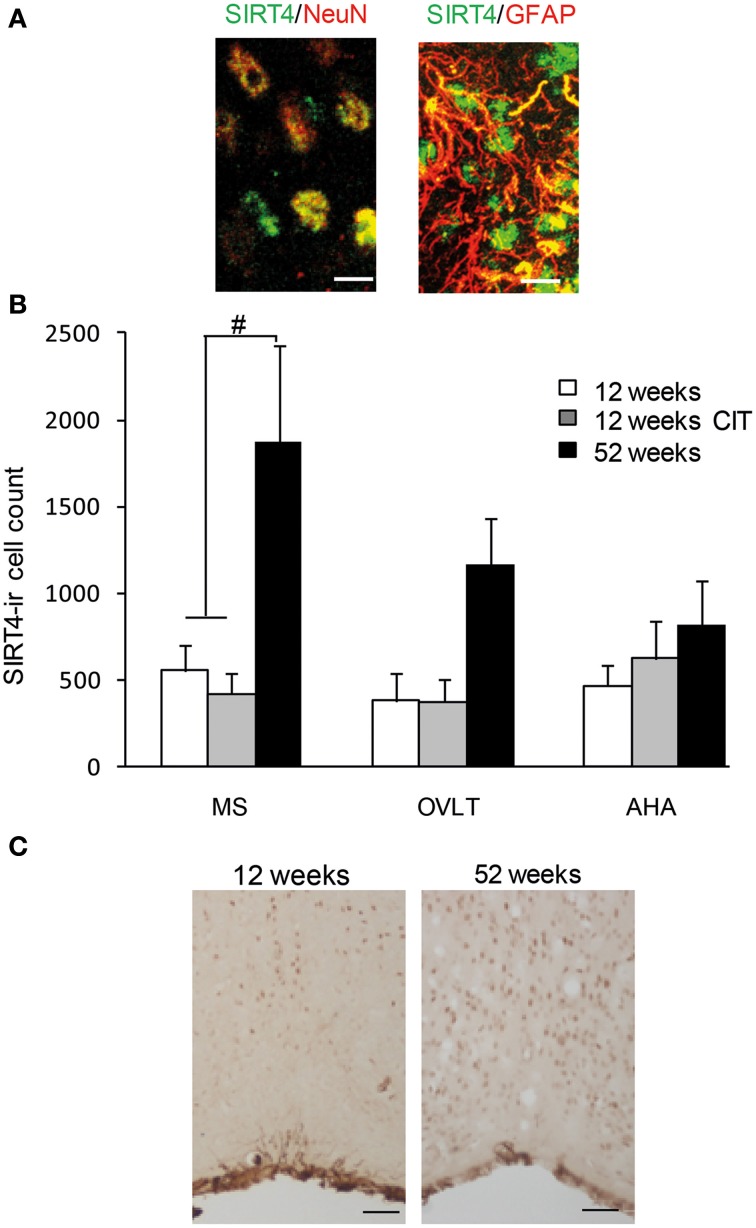
**Aging but not citalopram (CIT) induced SIRT4 immunoreactive cells in the medial septum (MS)**. **(A)** SIRT4 in the POA was observed to be highly co-localized with neuronal nuclei (NeuN) compared to glial fibrillary acidic protein (GFAP) Scale bar = 10 μm. **(B)** SIRT4 immunoreactive cell counts revealed up-regulation of SIRT4 during aging in the MS but not in the organum vasculosum of the lamina terminalis (OVLT) and anterior hypothalamic area (AHA). However, CIT treatment did not alter any SIRT4 immunoreactive cell counts. **(C)** High magnification image of SIRT4 staining in MS of 12 weeks and 52 weeks. Scale bar = 50 μm. Data are expressed as mean ± SEM. Statistical analysis was carried out using analysis of variance, ^#^*P* < 0.01 vs. control.

SIRT4 immunoreactive cells were observed in the MS, OVLT and AHA regions (Figure 4). We observed SIRT4 protein localized mainly in neurons compared to glial cells in the POA (Figure 5A). SIRT4 co-localization with glial cells were observed in areas close to the third ventricle of the preoptic area whereas the vast majority of SIRT4 immunoreactivity was observed in neurons. There was no immunostaining when the antibody was preadsorbed with its corresponding antigen or when the antibody was excluded.

There was an increase in SIRT4 immunoreactive cell counts in the MS of 52weeks aging male mice (12 weeks 421 ± 116, 12 weeks CIT 559 ± 146, 52 weeks 1521 ± 306) [Age effect in the MS, *F*_(1, 16)_ = 16.98, *p* < 0.01] and *T*-test revealed that SIRT4 immunoreactivity at 52 weeks was higher than 12 weeks (*p* < 0.05) and 12 weeks CIT treated mice (*p* < 0.01) (Figures 5B,C). CIT treatment did not alter SIRT4 immunoreactivity in the MS. Age and CIT treatment did not alter SIRT4 expression in the OVLT and AHA (Figure 5B).

## Discussion

In this study, we observed an age-related up-regulation of *sirt1, sirt4, sirt5*, and *sirt7* gene expression in the POA. The expression patterns of *sirt1, sirt4, sirt5*, and *sirt7* gene were different at 36 and 52 weeks, which suggest that different regulatory factors might be involved in their control. Higher levels of *sirt4* and *sirt5* mRNA at 52 weeks may be linked with a decrease in 5-HT (Hussain and Mitra, 2000) and a decrease in testosterone levels (Eleftheriou and Lucas, 1974; De Marte and Enesco, 1986) which are observed during the aging process. In contrast to *sirt4* and *sirt5* expression pattern in the POA, the expression level of *sirt7* was higher in 36 weeks than in 52 weeks old animals. This could be due to an overall decrease in transcription activity in the brain at 52 weeks or due to hypothalamic neurodegeneration that begins at 36 weeks (Bourre and Piciotti, 1992). Since *sirt1* is an age induced gene (Duan, 2013), the increase expression of *sirt1* in the POA of 36 weeks old animals was not unexpected. *Sirt 1* gene expression may be regulated by site-specific modulation in the brain. As *sirt* 1 gene is a regulator of metabolic functions, centrally through the hypothalamus (Duan, 2013), up-regulation of sirt1 gene expression may be due to metabolic changes at 36 weeks in the POA.

CIT treatment up-regulated only *sirt2* and *sir4* gene in the POA. Sirt1 remained unchanged following CIT treatment as it does not respond to SSRI-class of antidepressant (Kishi et al., 2011). This suggests that the effect of CIT on *sirt1* might involve factors aside from 5-HT that change in the male HPG axis during aging (Veldhuis, 2008). CIT treatment may affect *sirt2* gene through 5-HT signaling in the POA, since *sirt2* gene up-regulation is also seen in patients during remission state of depression (Abe et al., 2011). On the other hand, *sirt4* gene expression is regulated by age and CIT in the POA. A decrease in 5-HT during aging (Hussain and Mitra, 2000) has been linked with neurodegenerative diseases (Glorioso et al., 2011), decreased gonadotropin release and cognitive loss (Alzheimer's disease) (Simpkins et al., 1977; Meltzer et al., 1998). Antidepressants like CIT and fluvoxamine cause sexual dysfunction in rodents (Montejo et al., 2001; Soga et al., 2010), and human (Waldinger et al., 2001). Recent studies have shown that chronic SSRI treatment could decrease 5-HT content and signaling in the brain (Delgado et al., 1990; Hervás and Artigas, 1998), rather than simply facilitating synaptic 5-HT availability (de Jong et al., 2006; Geddes et al., 2015). Therefore, age-related and CIT induced sexual dysfunction and cognitive loss due to the decline in 5-HT in the POA may be mediated by an up-regulation of *sirt4* gene expression.

**Figure 6:**
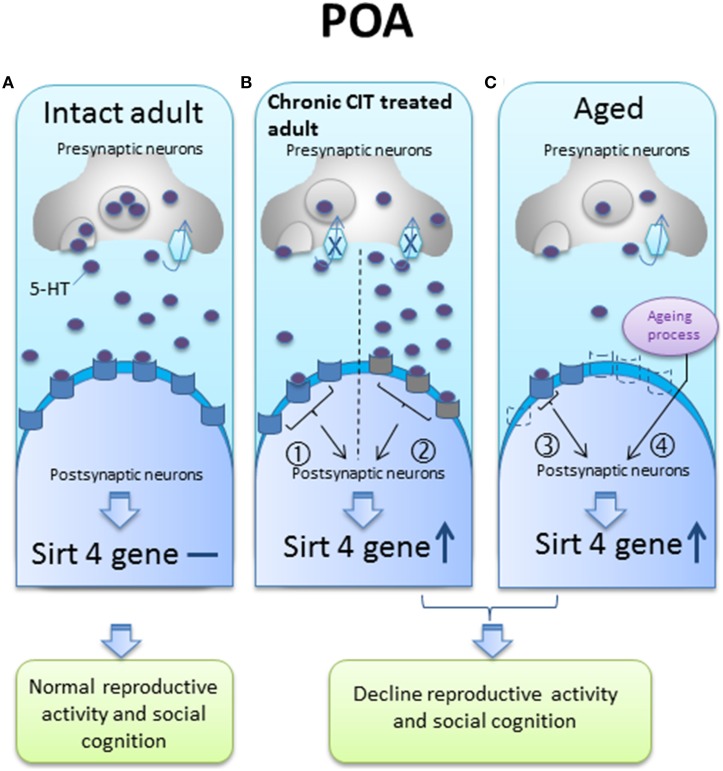
**A hypothetical diagram of sirt4 gene regulation by serotonin (5-HT) in the preoptic area (POA) of intact adult, chronic citalopram (CIT) treated adult and aged animals**. **(A)** In the intact adult, sirt4 gene expression levels do not change; **(B)** in the chronic CIT treated adult, the inhibition of 5-HT re-uptake results in decreased presynaptic 5-HT content (pathway ①) or desensitization of postsynaptic receptors that could subsequently be less activated in the presence of normal, or even increased, synaptic 5-HT concentrations (pathway ②), either of which can result in decreased 5-HT neurotransmission and increased sirt4 gene expression; **(C)** in aged animals, the serotonergic system may be impaired by decreased 5-HT synthesis (pathway ③) or a decrease in postsynaptic 5-HT receptors (dotted receptor) (pathway ④) to explain sirt4 upregulation, or an unknown age related effect could increase sirt4 gene expression. These 5-HT signaling pathways could facilitate sirt4 gene expression in the POA leading to reproductive and cognitive failure. 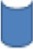 5-HT receptors 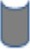 Desensitized 5-HT receptors 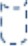 Decrease 5-HT receptors 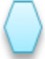 5-HT transporter 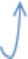 re-uptake 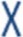 Inhibition of re-uptake 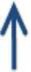 increase 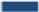 No change in gene expression, ① decrease 5-HT neurotransmission, ② desensitized receptor, ③ decrease 5-HT neurotransmission, ④unknown age-related factors could activate sirt4 gene expression.

We speculate that the up-regulation of sirt4 gene expression in the POA might occur through one or several potential mechanisms, such as: (1) the blockade of 5-HT uptake by chronic CIT treatment could decrease presynaptic 5-HT content or desensitize postsynaptic 5-HT receptors; (2) the aging process might be associated with a decrease in 5-HT synthesis or a decline in postsynaptic 5-HT receptors (Figure 6). According to our model, any of these events could explain a change in sirt4 expression associated with a decrease in serotonergic tone.

The localization of SIRT4 primarily in adult neurons is similar to other members of the SIRT family; SIRT1 and SIRT2 in neurons (Houtkooper et al., 2012). The roles of SIRT1, 2 and 4 in neuronal and glial development have been reported (Prozorovski et al., 2008; Park et al., 2012; Komlos et al., 2013). SIRT4 might function in tandem with other SIRT during early development of glial cells and aging of neurons in the POA. Unlike the increase in *sirt4* mRNA, our failure to observe an increase in the number of SIRT4 immunostained cells in the POA following CIT treatment could be due to the methodology used to detect protein levels. Although, immunohistochemistry is an accepted semi-quantitative measure of protein levels; a subtle change in protein levels within cells could go undetected using immunocytochemistry and thereby result in unaltered SIRT4 cell numbers.

The POA is involved in social cognitive functions such as paternal behavior, social recognition and reproductive behavior (Ferguson et al., 2002). An age-related decline in cognitive and reproductive functions (Meltzer et al., 1998) might be associated with decline in the serotonergic system. The POA is known to project to brain regions important for cognitive functions such as the dorsal raphe that harbors 5-HT neurons and the hippocampus (Sava and Markus, 2008). This suggests that the anatomical and functional connection between the POA and the hippocampus and the dorsal raphe might be involved in age-related cognitive impairment. Inversely, 5-HT neurons are known to project to GnRH neurons in the POA (Jennes et al., 1982). A decrease in 5-HT during aging could decrease GnRH levels through the activation of sirt, which could cause a decrease in LH receptors and GnRH receptors in the hippocampus, resulting in deregulation of the social cognitive functions.

Hence, we suggest that the age-related and CIT-induced activation of *sirt4* gene expression, might be initiated by the decline in 5-HT in the POA which leads to reproductive dysfunction and cognitive deficits.

### Conflict of interest statement

The authors declare that the research was conducted in the absence of any commercial or financial relationships that could be construed as a potential conflict of interest.

## Supplementary material

Supplementary Table 1 **Sequences of primers used for real-time PCR**.
