# Supplementary material for: Aging and chronic administration of serotonin-selective reuptake inhibitor citalopram upregulate Sirt4 gene expression in the preoptic area of male mice
Source: Front Genet. 2015 Sep 16;6:281. doi: 10.3389/fgene.2015.00281 (PMC4584971; doi:10.3389/fgene.2015.00281)
Supplement: Supplementary Table 1 — Sequences of primers used for real-time PCR. [file Table1.PDF]

Primer	Sequence	Product size	Accession number
Cyclophilin-Forward	5'-TGCTGG ACCAAACACAAACGGTTC-3'	129bp	NM_008907.1
Cyclophilin-Reverse	5'-AAACGCTCCATGGCTTCCACAATG-3'		
sirt1-Forward	5'-CACCAAAGCGGAAAAAAAGAAA-3'	104bp	NM_019812
sirt1-Reverse	5'-AGAAACCCCAGCTCCAGTCA-3'		
sirt2-Forward	5'-CAAGCCAACCATCTGCCACTA-3'	102bp	NM_022432
sirt2-Reverse	5'-CCCGCCACTCGTTCCA-3'		
sirt3-Forward	5'-GCTGCTTCTGCGGCTCTATAC-3'	231bp	NM_022433
sirt3-Reverse	5'-TGCTCCCCAAAGAACACAATG-3'		
sirt4-Forward	5'-GTCCCGTGCTGTGATCGA-3'	179bp	NM_001167691
sirt4-Reverse	5'-CGGGCGGTGAGGATGAAC-3'		
sirt5-Forward	5'-GCCTCCCCACAAAGCAAGA-3'	135bp	NM_178848
sirt5-Reverse	5'-AACCCCACTCTCCGCACTAA-3'		
sirt6-Forward	5'-CCTGCCCCTTGCCACTAA-3'	118bp	NM_181586
sirt6-Reverse	5'-GCACATCACCTCATCCACGTA-3'		
sirt7-Forward	5'-GCCAGGAGGAGGTGTGTGA-3'	214bp	NM_153056
sirt7-Reverse	5'-GGCTCCGCTTCGCTTAGGT-3'		
